# Crystal Structure of a Charge Engineered Human Lysozyme Having Enhanced Bactericidal Activity

**DOI:** 10.1371/journal.pone.0016788

**Published:** 2011-03-07

**Authors:** Avinash Gill, Thomas C. Scanlon, Daniel C. Osipovitch, Dean R. Madden, Karl E. Griswold

**Affiliations:** 1 Thayer School of Engineering, Dartmouth College, Hanover, New Hampshire, United States of America; 2 Program in Experimental and Molecular Medicine, Dartmouth College, Hanover, New Hampshire, United States of America; 3 Department of Biochemistry, Dartmouth Medical School, Dartmouth College, Hanover, New Hampshire, United States of America; 4 Program in Molecular and Cellular Biology, Dartmouth College, Hanover, New Hampshire, United States of America; 5 Department of Biological Sciences, Dartmouth College, Hanover, New Hampshire, United States of America; Institut Pasteur, France

## Abstract

Human lysozyme is a key component of the innate immune system, and recombinant forms of the enzyme represent promising leads in the search for therapeutic agents able to treat drug-resistant infections. The wild type protein, however, fails to participate effectively in clearance of certain infections due to inherent functional limitations. For example, wild type lysozymes are subject to electrostatic sequestration and inactivation by anionic biopolymers in the infected airway. A charge engineered variant of human lysozyme has recently been shown to possess improved antibacterial activity in the presence of disease associated inhibitory molecules. Here, the 2.04 Å crystal structure of this variant is presented along with an analysis that provides molecular level insights into the origins of the protein's enhanced performance. The charge engineered variant's two mutated amino acids exhibit stabilizing interactions with adjacent native residues, and from a global perspective, the mutations cause no gross structural perturbations or loss of stability. Importantly, the two substitutions dramatically expand the negative electrostatic potential that, in the wild type enzyme, is restricted to a small region near the catalytic residues. The net result is a reduction in the overall strength of the engineered enzyme's electrostatic potential field, and it appears that the specific nature of this remodeled field underlies the variant's reduced susceptibility to inhibition by anionic biopolymers.

## Introduction

Chronic pulmonary infections are a major cause of patient morbidity and mortality in diseases ranging from cystic fibrosis (CF) to chronic obstructive pulmonary disease (COPD) and pneumonias. In CF, polymicrobial airway infections are established early, and by adulthood most patient airways are persistently colonized by the opportunistic pathogen *Pseudomonas aeruginosa*, the primary cause of patient mortality [1]. Although a complete understanding of CF pathology remains elusive, it is thought that increased viscoelasticity of the airway surface liquid and reduced mucociliary clearance facilitate the establishment of chronic bacterial infections [2]. In addition to physiological factors that favor the persistent nature of CF infections, drug-resistance is a critical issue for both Gram-negative *P. aeruginosa* [3], [4] and Gram-positive pathogens such as *Staphylococcus aureus* [5] and various streptococci [6]. To more effectively manage bacterial infections associated with CF and other diseases such as COPD and pneumonias, there is a critical need for next generation antibiotics capable of treating drug-resistant pathogens. In one approach to new therapies, genetically engineered antimicrobial proteins are being developed based on knowledge of the mechanisms by which innate immune factors sometimes fail.

Human lysozyme (hLYS) kills bacteria by catalytic hydrolysis of cell wall peptidoglycan, but also exhibits catalysis-independent antimicrobial properties [7]. Its dual functions result in a protein that attacks both Gram-positive and Gram-negative bacterial pathogens, and hLYS has been shown to be the most effective cationic anti-pseudomonal agent in human airway fluids [8], [9]. In principle, this antimicrobial profile suggests that recombinant hLYS could serve as a potent, protein therapeutic if delivered to the airway using inhalation technologies such as those developed for the FDA-approved, DNA-degrading enzyme Pulmozyme [10]. However, the failure of endogenous hLYS to effectively clear bacteria during chronic infections indicates that the wild type sequence suffers from some specific dysfunction in the infected lung environment. Understanding and mitigating the inherent functional limitations of wild type hLYS could facilitate development of novel, antimicrobial, enzyme therapies.

The cationic nature of hLYS is thought to play an important role in guiding the protein to the negatively charged surface of bacteria. The dense positive charge of hLYS, however, also represents an Achilles' heel, as the wild type enzyme can be sequestered and inactivated by alginate [11], a biofilm matrix component associated with mucoid *P. aeruginosa* lung infections [12]. Furthermore, lower respiratory tract infections drive a hyperinflammatory immune response, and subsequently cause the local accumulation of additional, densely charged, anionic biopolymers including F-actin, DNA, and mucin [13], [14]. In the infected lung, these biopolymers may exceed 1% wt/vol. Concentrated polyanions radically alter the electrostatic environment of airway surface liquid, and are thought to inhibit various cationic antimicrobial peptides and proteins [15]. This type of electrostatic sequestration has been experimentally demonstrated with hen egg white lysozyme [16], and variants of T4 phage lysozyme having fewer cationic residues exhibit a reduced propensity to complex with F-actin while retaining ∼50% antibacterial activity in phosphate buffered saline (PBS) [17]. Building upon these studies, we sought to develop genetically engineered lysozyme variants designed specifically for high level activity in the presence of various disease-associated, anionic biopolymers, and against both Gram-negative and Gram-positive bacterial species.

## Results and Discussion

### Enhanced Catalytic Function

**Figure 1:**
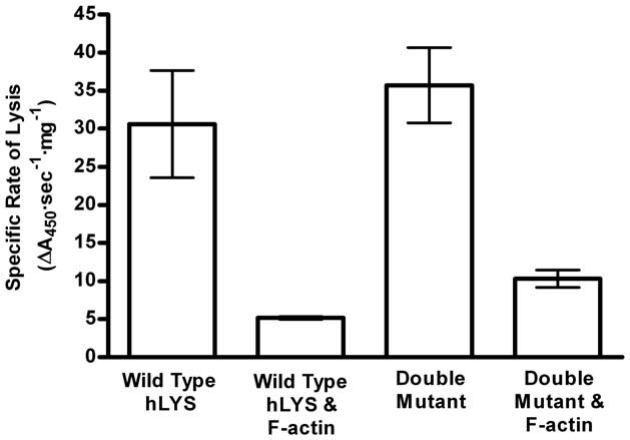
Lysozyme Inhibition by F-actin. In the absence of inhibitor, the double mutant lyses bacteria at a rate equivalent to wild type hLYS, but in the presence of inhibitory F-actin the kinetics of the engineered enzyme are 2-fold faster (p = 0.001, two tailed t-test).

In an effort to reduce the immunogenic potential of our prospective therapeutic enzymes, we employed a human protein scaffold as a starting template. Combinatorial libraries of charge engineered hLYS variants were designed using bioinformatics and structural analysis, and approximately 150,000 mutated enzymes were screened for bacteriolytic activity in the presence of inhibitory alginate polyanion. Among other functionally enhanced enzymes, the Arg101→Asp and Arg115→His double mutant was found to lyse bacteria effectively at alginate, mucin and DNA concentrations that inactivated wild type hLYS. Moreover, in the absence of inhibitory biopolymers, the mutations did not substantially impair the enzyme's *V_max_* or *K_m_*, had no effect on its *in vitro* anti-pseudomonal activity, and did not reduce lytic function [11]. Indeed, time course killing assays in a standard lysozyme activity buffer (66 mM phosphate, pH 6.24) revealed that the double mutant's non-inhibited kinetics were faster than those of wild type hLYS [11]. More recently, we have extended the inhibition assays to include actin, which is known to be a key inhibitor of therapeutic proteins relevant to lung infections [14], [17]. In these studies, we chose to focus on the Gram-positive lytic activity of hLYS, and we therefore employed the model organism *Micrococcus luteus*. Our kinetic analysis is the first direct, experimental demonstration that charge engineering can enhance lysozyme activity *in the presence* of inhibitory F-actin (Fig. 1). Thus, combinatorial mutation of hLYS combined with high throughput functional screening generated an enzyme variant with decreased net charge, reduced susceptibility to anionic biopolymer inhibition, and *no* loss of intrinsic bacteriolytic activity.

### Structural and Sequence Analysis

**Figure 2:**
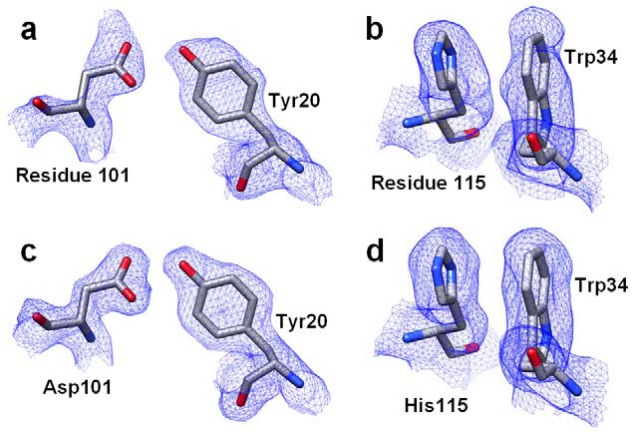
Experimental Electron Density for the Double Mutant Lysozyme. (Top Panels) Iterative Build Composite Omit Maps used for initial model building. (a) Observed electron density for residue 101 and neighboring residue Tyr20 shown as blue wire mesh. (b) Observed density for residue 115 and neighboring residue Trp34. Residues are shown as stick figures placed in the electron density omit maps: Carbon-grey; Nitrogen-blue; Oxygen-red. The amino acids were placed in the model when clear electron density was observed at these positions in the omit map. (Bottom Panels) Final refined electron density maps for (c) Asp101 & Tyr20 and (d) His115 & Trp34. Figure rendered with CHIMERA [31].

To gain molecular level insight into the double mutant's enhanced function, we determined the protein's X-ray crystal structure. Analysis of the Matthews coefficient [18] suggests that there are 2 molecules per asymmetric unit, with a V_M_ value of 2.33 Å^3^ Da^−1^. We selected a mutation-free hLYS structure, 1LZS, as a search model for molecular replacement studies, and subsequently identified a clear solution containing two protomers. To avoid model bias associated with the high degree of sequence identity between the wild type protein and the double mutant (98.5%), iterative-build composite omit maps were calculated (Fig. 2). The high quality electron density permitted direct modeling of the initial molecular replacement solution. The mutant residues at positions 101 and 115 of the protein sequence search model were initially defined as alanine residues. Side chains for Asp101 and His115 were substituted only after observing clear electron densities at the respective positions. Iterative model building and refinement to 2.04 Å resolution yielded a unit cell containing 2050 atoms in two protein chains and 237 water molecules, which was in excellent agreement with experimental diffraction data (Table 1).

**Table 1:** Data Collection and Refinement Statistics.

**Data Collection**	
Space Group	*P*2_1_2_1_2_1_
Unit Cell Parameters (Å, °)	42.42 63.79 111.08, 90 90 90
Matthews coefficient (Å^3^/Da)	2.33
Solvent content (%)	46.9
Resolution^a^	19.86–2.04 (2.14–2.04)
Total reflections^a^	134735 (16297)
Unique reflections^a^	19829 (2606)
R_sym_ (%)^a^ ^b^	6.5 (18.6)
Completeness^a^(%)	99.7 (99.5)
I/σI^a^	21.6 (9.5)
**Refinement Statistics**	
# protein atoms (non-H)	2050
# waters	237
R_work_ (%)^a^	18.1 (17.7)
R_free_ (%)^a^	22.2 (22.9)
Ramachandran plot^c^ (%)	97.7/2.3/0
RMSD (Bonds/Angles) (Å, °)	0.008/1.014
Average Isotropic B-factors (Å^2^)	
Main chain (A/B)^d^	14.91/15.08
Side Chain (A/B)^d^	18.36/17.64
Waters	22.43

a Values in parentheses indicate statistics for the highest resolution shell of data;

b R_sym_ = Σ_hj_|Î_h_−I_hj_|/Σ_hj_I_hj_;

c Favored/allowed/outliers;

d Residual B factors (does not include the contribution to atomic displacements from translation, libration and screw-rotation displacement).

**Figure 3:**
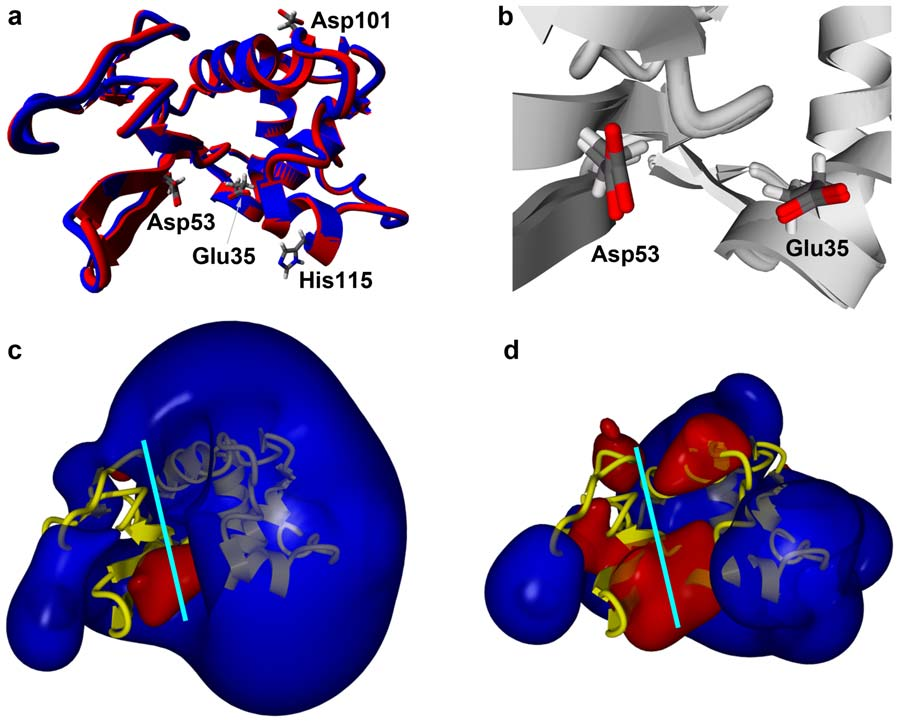
Structural Comparison of Double Mutant and Wild Type hLYS. (a) Overlaid ribbon diagrams of the peptide backbones; wild type hLYS in blue (PDB file 1JWR) [32] and charge engineered double mutant in red (PDB file 3LN2). The catalytic residues (35 and 53) and the two mutated residues of 3LN2 (101 and 115) are shown as stick models with: Carbon-grey; Hydrogen-white; Nitrogen-blue; Oxygen-red. The structural alignment yielded a low root-mean-squared (RMS) deviation of 0.42 Å for the backbone atoms and 1.17 Å overall. (b) Detailed view of the active site indicating negligible differences in the position of the catalytic residues Glu35 and Asp53. (c) The electrostatic potential field of wild type hLYS contoured as a 110 kJ/mol surface. Calculation performed with the AMBER99 force field in an 83×70×77 Å simulation cell with periodic boundaries. Positive potential is colored dark blue and negative potential red. The peptide backbone is rendered as a yellow ribbon, and the substrate binding cleft is indicated with a light blue line. The field is overwhelmingly positive in nature with negative potential localized predominantly near the catalytic residues Glu35 and Asp53. (d) The analogous electrostatic potential field of the double mutant contoured at 110 kJ/mol. The global electrostatic field is contracted relative to the wild type protein, as seen by the reduced size of the 110 kJ/mol surface. Additionally, the potential of the active site cleft has been extensively remodeled, and exhibits several expanded regions of negative potential. A small region of positive potential at the upper lip of the active site cleft is maintained at wild type strength (blue lobe at left), as is a larger portion of positive potential distal to the active site cleft (protruding lobe at far right of image). Calculations performed and images rendered with YASARA Structure v9.10.5.

**Figure 4:**
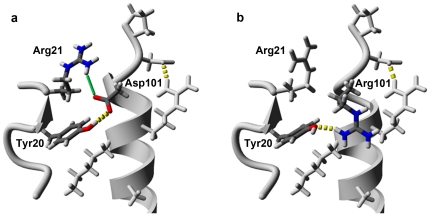
Residue 101 Interaction Analysis. A partial ribbon diagram of the peptide backbone is shown with neighboring side chains rendered as stick figures. Residue 101 and interacting partners are differentially colored: Carbon-grey; Hydrogen-white; Nitrogen-blue; Oxygen-red. (a) The local environment of Asp101 in the double mutant structure (PDB file 3LN2). At image center, a carboxylate oxygen of Asp101 is 3.36 Å from a guanidino amine of Arg21 resulting in an electrostatic N-O bridge (green line) [19]. At the same time, the Asp101 side chain is within hydrogen bonding distance of Tyr20's phenolic group (broken yellow line, 2.67 Å O-O distance). (b) The local environment of Arg101 in wild type hLYS (PDB file 1JWR) [32]. At image center, the wild type Arg101 is able to hydrogen bond with Tyr20 (broken yellow line, 2.92 Å N-O distance), but orients away from Arg21 as a result of electrostatic repulsion. Images rendered with YASARA Structure v9.10.5.

The Arg101→Asp and Arg115→His mutations result in minimal perturbation of the global enzyme structure, as demonstrated by a superposition of the double mutant model onto the crystal structure of wild type hLYS (Fig. 3a). The mutated residues are situated at opposite ends of the substrate-binding groove, and as a result they have little impact on the catalytic core of the active site (Fig. 3b). Both mutated residues are stabilized by conformation specific interactions with native amino acids that are adjacent in space but distant in the primary sequence. The mutant Asp101 side chain forms an electrostatic N-O bridge [19] with Arg21, and is within hydrogen bonding distance of Tyr20 (Fig. 4a). Although the wild-type Arg101 side chain also forms a hydrogen bond with Tyr20, it does so with a geometry different from the mutant Asp101. Even more striking, repulsive charge-charge interactions cause the wild type Arg101 and neighboring Arg21 side chains to orient away from each other (Fig. 4b). At the second mutation site, the His115 side chain lies alongside Trp34 in a parallel displaced π-stacking interaction [20] (Fig. 2d). This orientation is similar to that of the wild type Arg115 side chain, and reproduces an interaction found among natural lysozymes having a histidine at this position, e.g. see canine milk lysozyme (PDB code 1QQY [21]).

A Consurf bioinformatics analysis [22] of 50, naturally occurring, C-type lysozyme orthologs was crucial in the development of the highly active double mutant [11], and a close examination of the results places the two performance-enhancing mutations within the context of natural lysozyme sequences. Using a multiple sequence alignment generated with the MUSCLE algorithm [23] (Fig. S1), the natural sequence variability of individual amino acid positions was assessed for the 50 lysozyme orthologs. A normalized evolutionary conservation score was then calculated for each of hLYS's 130 residues. The most highly conserved 25% of residues scored between −1.026 and −0.906, whereas the most poorly conserved 25% of residues scored between 0.589 and 2.811 (Fig. S2). In the multiple sequence alignment, residue 101 is occupied by an arginine in 24 cases, a serine in 24 cases, and a leucine or gap in the remaining two enzymes (Fig. S1). Despite the limited amino acid diversity at position 101, the lack of a clear consensus translates to a low degree of evolutionary conservation (normalized score = 0.895, least-conserved quartile). Significantly, the Asp101 side chain of our highly active double mutant forms multiple stabilizing interactions with neighboring amino acids (Fig. 4a), even though this residue does not naturally occur at position 101 among orthologs of our bioinformatics analysis (Fig. S1).

In contrast to position 101, position 115 was a consensus histidine in 38 of the lysozyme orthologs. In addition to the 38 enzymes possessing a consensus histidine, four orthologs encode Lys115, four Arg115, two Trp115, one Asn115, and one Gln115 (Fig. S1). The comparatively large stereochemical diversity among alternative residues at this site resulted in a low degree of evolutionary conservation (normalized score = 0.733, least-conserved quartile) similar to that of position 101. Notably, our highly active double mutant substitutes the wild type Arg115 with a consensus histidine. It is therefore not surprising that the mutant His115 side chain exhibits an energetically favorable interaction with at least one neighboring side chain (Fig. 2d).

### Electrostatic Potential Analysis

The propensity for wild type hLYS to complex with and be inhibited by anionic biopolymers is due in large part to Coulombic interactions, and as expected the functionally enhanced double mutant has reduced cationic character. The calculated isoelectric point of wild type hLYS is 9.3, and its predicted net charge at neutral pH is +7.8 (Vector NTI sequence analysis software, Invitrogen, Carlsbad, CA). The corresponding values of the double mutant are 8.9 and +4.9, respectively. Thus, the two mutations should result in a substantial reduction in net charge near physiological pH, although the proteins were not clearly resolved on pH 3–10 isoelectric focusing gels (data not shown). To visualize the basis for the double mutant's enhanced performance in greater detail, we calculated the molecule's electrostatic potential field and compared the result to an identical analysis of the wild type structure. Consistent with the net charge shift, the overall strength of the double mutant's potential field is substantially decreased relative to wild type hLYS. At the same time, the two mutations manifest an expanded negative potential field in and around the substrate binding cleft (compare Fig. 3c and 3d). Despite these considerable changes, small portions of the double mutant's positive potential field have been maintained at wild type strength.

### Stability Analysis

The structural stability of therapeutic proteins can play a key role in (i) preventing aggregation during storage and (ii) enhancing efficacy by preventing *in vivo* degradation. We therefore sought to assess the extent to which the Arg101→Asp and Arg115→His mutations influenced lysozyme stability. Thermal denaturation studies of the double mutant and wild type enzymes were conducted by differential scanning fluorimetry [24]. In PBS pH 7.4, wild type hLYS had a T_m_ = 63.7±0.6°C and the engineered double mutant had a T_m_ = 63.8±0.7°C. Thus, the two mutations had no significant effect on the engineered enzyme's thermal stability (p = 0.45).

**Figure 5:**
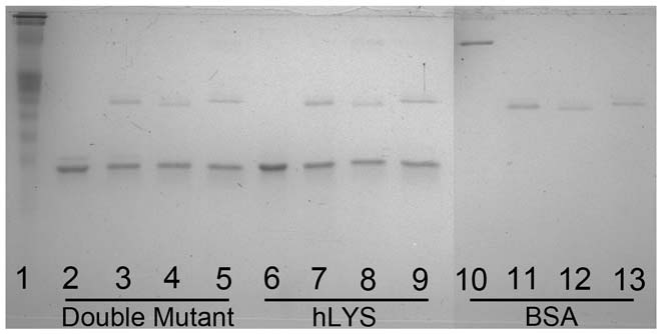
Proteolytic Susceptibility Analysis of Lysozymes. An SDS-PAGE gel was Coomassie stained for total protein. Lane 1 = Kaleidoscope prestained molecular weight standards. Lanes 2–5 = 900 ng of the engineered double mutant. Lane 2 is an untreated control, and lanes 3, 4, and 5 are 50 µg/ml treatments with human neutrophil elastase, cathepsin G, and proteinase 3, respectively. Lanes 6–9 are the wild type hLYS arrayed in a similar fashion. Lanes 10–13 are bovine serum albumin (BSA) arrayed in a similar fashion. The wild type hLYS and engineered double mutant migrate just below the 15 kDa band as expected (lanes 2–9). The BSA control migrates between the 50 and 75 kDa bands as expected (lane 10). The three proteases can be seen migrating between the 25 and 37 kDa bands in the treatment lanes. Neither wild type hLYS nor the double mutant is degraded by the proteases, while BSA is fully degraded by all three. Similar results were obtained with both 5 µg/ml and 0.5 µg/ml protease treatments (data not shown), although BSA was only partially degraded by 0.5 µg/ml proteinase 3.

In addition to thermal stability, resistance to human neutrophil proteases is an important parameter for pulmonary biotherapies, as neutrophil derived proteases can accumulate to micromolar concentrations in the infected and inflamed lung [25]. We therefore assessed proteolysis of the double mutant and wild type hLYS by human neutrophil elastase, cathepsin G, and proteinase 3. Our analysis shows that neither enzyme is susceptible to degradation by physiologically relevant concentrations of the three neutrophil proteases (Fig. 5). Combined with the thermal denaturation studies, these results indicate that the Arg101→Asp and Arg115→His mutations do not alter the double mutant's structural stability relative to wild type hLYS.

### Conclusion

The double mutant detailed in this manuscript is not the only functionally enhanced enzyme to be isolated in the course of large combinatorial library screens. More than 30 different charge modified variants having diverse sequences, net charges, and charge distributions were found to exhibit greater lytic activity than wild type hLYS in the presence of inhibitory alginate biopolymer [11]. The Arg101→Asp and Arg115→His double mutant, however, is the only variant thus far proven to retain wild type or better lytic activity in the *absence* of inhibitory biopolymers (Fig. 1). This observation underscores the complex interplay between hLYS charge distribution and bacteriolytic function, and it suggests that specific regions of stereochemical conservation are key to engineering lysozymes with high level activity in both inhibitory and non-inhibitory conditions.

In summary, we have engineered the first lysozyme protein specifically designed for enhanced function in the presence of disease-associated, anionic biopolymers. This biotherapeutic candidate kills both Gram-negative (*P. aeruginosa*) and Gram-positive (*M. luteus*) bacteria more efficiently than the wild type protein under inhibitory conditions, and our structural analysis indicates that a reshaped electrostatic potential field underlies its enhanced antibacterial properties. The substituted amino acids of the variant enzyme exhibit energetically favorable interactions with spatially adjacent amino acids, and as a result the global structure and stability of the variant are equivalent to those of the wild type protein. While the charge engineered hLYS variant is particularly relevant to CF-associated bacterial infections, its broad spectrum utility may ultimately extend to other chronic or acute lung infections resulting from COPD, hospital acquired pneumonia or community acquired pneumonia.

## Materials and Methods

Recombinant hLYS, freeze-dried *M. luteus*, and SYPRO Orange 5000× Protein Stain were purchased from Sigma-Aldrich (St. Louis, MO). MicroAmp® Fast Optical 0.1 ml 96-Well Plates and MicroAmp® Optical Adhesive Film were from Applied Biosystems (Bedford, MA). Human neutrophil elastase, human neutrophil cathepsin G, and human neutrophil proteinase 3 were from Athens Research & Technology (Athens, GA). Rabbit muscle G-actin was a kind gift from Dr. Henry N. Higgs and Ernest G. Heimsath, Jr. of Dartmouth College. All other chemicals and reagents were purchased from Fisher Scientific (Pittsburgh, PA).

### Lysozyme Inhibition Assay

Time course lysis of *Micrococcus luteus* bacteria was monitored by light scattering at 450 nm, and specific rates of lysis with and without F-actin are reported. Light scattering was monitored in clear bottom 96-well plates on a Molecular Devices SpectraMax 190 UV/Vis microplate spectrophotometer. Non-inhibited reactions contained 300 µg/ml *M. luteus* and 200 ng/ml lysozyme in 150 µl of 5 mM Tris pH 7.0. Reactions with F-actin were assembled as follows: 120 µl solutions of rabbit muscle G-actin were polymerized by agitating at 25°C for 15 minutes in 50 mM KCl, 2 mM MgCl_2_, 0.2 mM CaCl_2_, 1 mM ATP, and 5 mM Tris pH 7.0. Thirty nanograms of purified lysozyme in 5 µl of 5 mM Tris pH 7.0 were then added to the F-actin solutions, and agitation was continued for an additional 20 minutes. Lytic reactions were initiated by adding 25 µl of 1.8 mg/ml *M. luteus* stock solution. The final actin concentration in the lytic reactions was 836 µg/ml. All lytic rates were determined from slopes of the initial, linear portions of light scattering vs. time data. Reactions were performed in triplicate, and repeated experiments yielded essentially identical results. The double mutant kinetics were also 2-fold faster than wild type hLYS in F-actin reactions containing 333 ng/ml lysozyme (data not shown).

### Thermal Denaturation Studies

Differential Scanning Fluorimetry was performed essentially as described [24] using an ABI 7500 Fast Real-Time PCR System from Applied Biosystems (Bedford, MA). Proteins and SYPRO Orange were diluted in PBS. Final protein concentrations were 100 µg/ml and 50 µg/ml, and final dye concentrations were 20×, 10×, and 5×. Twenty µl reactions of all conditions (6 per protein) were performed in triplicate. The PCR gradient was run from 25–94°C with a 1 minute equilibration at each degree. Fluorescence was quantified using the preset TAMRA parameters. Melting temperatures were determined by data analysis with the “DSF Analysis v3.0.xlsx” Excel sheet (ftp://ftp.sgc.ox.ac.uk/pub/biophysics/) and GraphPad Prism v. 4 software (La Jolla, CA).

### Proteolytic Sensitivity Assays

Wild type hLYS, the purified double mutant, or BSA were diluted to 90 ng/µl in 10 µl reactions containing either elastase, cathepsin G, or proteinase 3. Three final protease concentrations were examined: 0.5 ng/µl, 5 ng/µl, and 50 ng/µl. All assays were conducted in PBS pH 7.4. Reactions were incubated for 3 hours at 37°C, and the products were immediately analyzed on 15% reducing, denaturing, SDS-PAGE gels. Bands were visualized with Code Blue gel stain.

### Crystallization and Data Collection

The isolation, expression, and purification of the mutated protein has been described in detail elsewhere [11]. Purified protein was dialyzed against crystallization buffer (10 mM KHPO_4_ pH 6.0, 100 mM NaCl) and concentrated to 7.0 mg/ml by centrifugal ultrafiltration. The protein was crystallized by 3–4 days of vapor diffusion against 20 mM sodium acetate pH 4.3, and 1.25 M NaCl at 18°C. The resulting crystals were cryoprotected by washing in mineral oil followed by soaking in well solution supplemented with 20% Ethylene Glycol. A complete dataset was collected for a single crystal at 100 K on a MAR345 image plate system using Cu Kα radiation produced with a rotating anode (Rigaku) equipped with focusing mirrors (Xenocs FOX-2D) and a Crystal Cryocooler (Cryo Industries).

### Model Building and Refinement

Data reduction, indexing, integration and scaling were done using programs of the XDS package [26]. Since the protein crystal exhibited non-crystallographic symmetry (NCS), the R_free_ set was selected in thin shells using the program SFTOOLS of the CCP4 package [27], and the same R_free_ dataset was used throughout refinement. Owing to the availability of numerous homologous hLYS structures, we used molecular replacement to obtain the initial phases. Wild type hLYS (PDB code: 1LZS [28]) was employed as a search model in the AutoMR program of the PHENIX software suite [29]. A clear solution was found with a log-likelihood gain of 2508 and a solvent content of 46.9%, as determined from the Matthew's coefficient calculation. The AutoBuild module of the PHENIX suite was used to calculate a density-modified, NCS-averaged, solvent-flattened, histogram-matched, iterative build omit map, and the initial model was built into this omit map manually using the program COOT [30]. The solution was then subjected to multiple rounds of refinement using the Refine module of PHENIX. This process included rigid body refinement, individual and grouped B-factor refinement, and NCS averaging. When the model was well-refined, water molecules were picked and refined. Any water molecules exhibiting weak electron density or instability during refinement were subsequently discarded. TLS refinement using each chain as a TLS group was carried out in the final stages of refinement.

### Protein Databank accession number

The atomic coordinates and structure factors of the double mutant have been deposited in the Protein Data Bank under accession number 3LN2.

## Supporting Information

Figure S1 **Multiple Sequence Alignment Generated by Consurf Analysis of PDB file 1LZS.** Wild type hLYS is the first sequence. Identical residues in the other lysozyme orthologs are noted with periods, and gaps are noted with dashes. Residues 101 and 115, the sites of mutation in this study, are boxed in red.
(PDF)

Figure S2 **Distribution of Consurf Evolutionary Conservation Scores for the Residues of Wild Type hLYS.** A bioinformatics analysis was conducted on PDB file 1LZS using the Consurf web server. The analysis generated position specific evolutionary conservation scores for each of the protein's 130 amino acids. Larger negative scores were assigned to highly conserved residues and larger positive scores to poorly conserved residues. The histogram shows the distribution of conservation scores for the entire hLYS sequence. The most poorly conserved 25% of residues scored between 0.59 and 2.81 (noted by bracket). Residues 101 and 115, the sites of mutation in the engineered variant, scored 0.895 and 0.733, respectively.
(TIF)
