# Supplementary material for: Crystal Structure of a Charge Engineered Human Lysozyme Having Enhanced Bactericidal Activity
Source: PLoS One. 2011 Mar 7;6(3):e16788. doi: 10.1371/journal.pone.0016788 (PMC3049763; doi:10.1371/journal.pone.0016788)
Supplement: Figure S1 — Multiple Sequence Alignment Generated by Consurf Analysis of PDB file 1LZS. Wild type hLYS is the first sequence. Identical residues in the other lysozyme orthologs are noted with periods, and gaps are noted with dashes. Residues 101 and 115, the sites of mutation in this study, are boxed in red. (PDF) [file pone.0016788.s001.pdf]

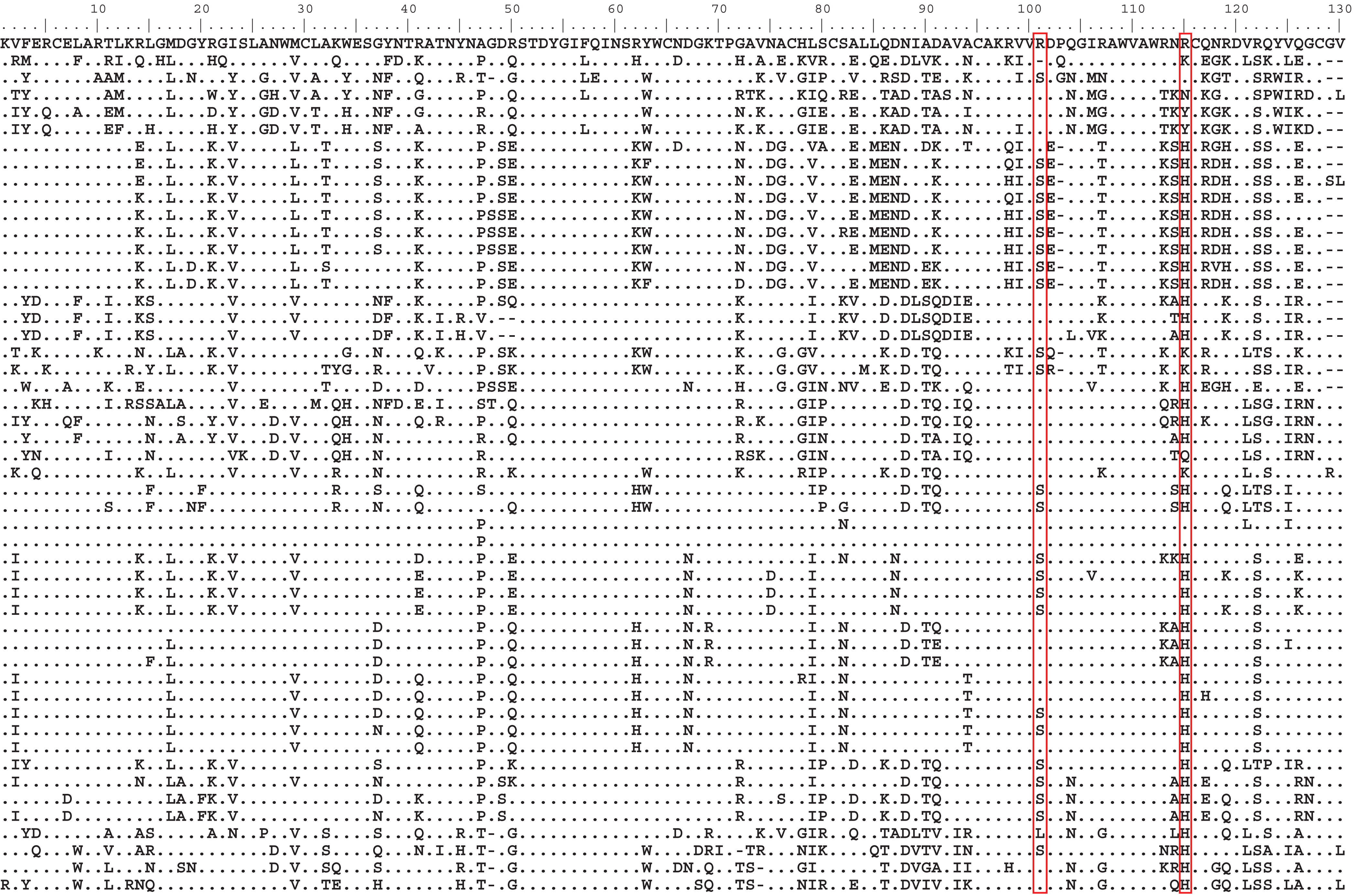

10 20 30 40 50 60 70 80 90 100 110 120 130
KVFERCELARTLKRLGMDGYRGISLANWMCLAKWESGYNTRATNYNAGDRSTDYGIFQINSRYWCNDGKTPGAVNACHLSCSALLQDNIADAVACAKRVVRDPQGIRAWVAWRNRCQNRDVRQYVQGCGV
